# Computable Structured Phenotype Versus Large Language Model Identification of Opioid Use Disorder Using Electronic Health Record Data

**DOI:** 10.1016/j.annemergmed.2026.05.005

**Published:** 2026-06-13

**Authors:** Melanie F. Molina, Cynthia Fenton, Kathy T. LeSaint, Samuel D. Pimentel, Michael A. Kohn, Aaron E. Kornblith

**Affiliations:** Department of Emergency Medicine (Molina, LeSaint, Kornblith), University of California, San Francisco, CA; Division of Clinical Informatics and Digital Transformation (Molina, Fenton), Department of Medicine, University of California, San Francisco, CA; Department of Statistics (Pimentel), University of California, Berkeley, CA; University of California, San Francisco, Department of Epidemiology and Biostatistics (Kohn), CA; Department of Pediatrics (Kornblith), University of California, San Francisco, CA.

**Keywords:** Large language models, Opioid use disorder, Screening, Clinical decision support, Structured computable phenotype

## Abstract

**Study objective:**

To compare a rule-based computable phenotype designed to identify patients with opioid use disorder in the emergency department (ED) with a large language model, using expert physician review as the reference standard.

**Methods:**

We conducted a retrospective study of randomly sampled adult ED encounters (January 1, 2023 to October 17, 2024) at a single academic health system. We drew a stratified random sample based on whether encounters met a preexisting rule-based phenotype for identifying opioid use disorder. The phenotype incorporated diagnosis codes, medications for opioid use disorder, urine toxicology results, addiction consultations, and keyword matching. With zero-shot prompting, a large language model (ChatGPT 4.1) classified opioid use disorder using ED notes from the index visit. Two board-certified emergency physicians independently determined the presence of opioid use disorder by full chart review; discrepancies were adjudicated by a third reviewer. Using inverse probability weighting based on the sampling fractions, we estimated sensitivity, specificity, positive predictive value, and negative predictive value.

**Results:**

Among 302 encounters, weighted opioid use disorder prevalence was 5.6% (95% confidence interval [CI], 4.0 to 7.0%). The structured phenotype demonstrated sensitivity 0.84 (95% CI, 0.42 to 0.97) and specificity 0.964 (95% CI, 0.96 to 0.97) (positive predictive value 0.58; negative predictive value 0.99). The large language model demonstrated sensitivity 0.81 (95% CI 0.70–0.88) and specificity 0.996 (95% CI, 0.993 to 0.998) (positive predictive value 0.92; negative predictive value 0.99). Specificity was significantly higher for the large language model (*P*<.0001).

**Conclusion:**

Both approaches demonstrated strong diagnostic performance. Although the structured phenotype showed slightly higher sensitivity, the large language model achieved higher specificity and positive predictive value, suggesting potential to reduce false-positive alerts in ED workflows. Prospective validation in other populations is needed.

## INTRODUCTION

By 2024, opioid use disorder affected more than 4.8 million Americans, and opioid-related overdoses accounted for more than 54,000 deaths, constituting a persistent public health crisis.^1,2^ The emergency department (ED) serves as a vital access point for patients with opioid use disorder, whether presenting with opioid use disorder-related or unrelated conditions.^3^ This creates an opportunity to connect patients to treatment and support.

Traditional approaches to identifying opioid use disorder in the ED include universal triage screening and inperson assessment by peer recovery specialists; however, these strategies are often inconsistently implemented due to competing clinical priorities, time constraints, and resource limitations.^4^ Failure to identify opioid use disorder during the ED visit represents a missed opportunity to initiate treatment and link patients to care.^5^ To address this challenge, prior work has developed computable structured opioid use disorder phenotypes—rule-based approaches that use structured electronic health record data (eg, diagnosis codes, medications, laboratory results) to identify patients likely to have opioid use disorder—to support screening and clinical decision-making.^6^ These approaches, validated across multiple health systems, classify patients based on predefined criteria rather than manual screening, enabling scalable, automated identification. In some settings, such phenotypes have been operationalized to activate real-time alerts or clinical decision support pathways to facilitate treatment, demonstrating feasibility for integration into routine ED workflows.^6^

However, computable structured opioid use disorder phenotypes may miss clinical nuance captured in narrative documentation and can generate false positives when structured data lack context. Recent advances in artificial intelligence (AI) offer additional promise for improving opioid use disorder identification, as large language models can mine unstructured clinical text for contextual information. Although AI approaches show promise for identifying opioid use disorder, their performance in ED settings, particularly large language models used without task-specific training, remains largely untested.^7^

As electronic health record vendors begin integrating large language model-based tools into ED clinical workflows, there is an increasing opportunity to evaluate whether they can support scalable opioid use disorder identification. However, integration of large language models into clinical care requires understanding their performance and limitations.

### Goals of This Investigation

We sought to compare a computable structured opioid use disorder phenotype against a large language model for opioid use disorder identification, using expert human determination as a reference standard.

## METHODS

### Study Design and Selection of Participants

We performed a retrospective analysis of electronic health record data extracted from the University of California, San Francisco (UCSF) Medical Center. The UCSF institutional review board approved the study. We included all ED encounters for patients aged 18 years and older between January 1, 2023 and October 17, 2024, reflecting the most recent period with complete electronic health record data at the time of extraction. Given opioid use disorder is relatively rare in our patient population, we used stratified sampling to generate an enriched sample of encounters for application of the reference standard. We first stratified encounters by a computable structured opioid use disorder phenotype currently implemented within our health system (described below).^8^ Within each of the 2 strata, we randomly selected one encounter per unique patient to avoid duplication, resulting in 302 patient-encounters: 201 meeting the structured opioid use disorder phenotype and 101 not meeting the phenotype. The sample size was selected to ensure at least 100 with opioid use disorder by the reference standard. Each included ED encounter was required to have associated ED notes available for large language model analysis; encounters without notes (eg, patients who left without being seen) were excluded.

### Measurements

#### Computable structured phenotype.

To identify vulnerable patients who could benefit from social work and care coordination, we used a rule-based computable structured phenotype to identify patients with clinical evidence of opioid use disorder from structured electronic health record data. We adapted a previously developed and validated structured opioid use disorder phenotype by Chartash et al^6^ for our local implementation as part of an ED-based clinical decision support tool that alerts clinicians to patients with possible opioid use disorder and facilitates treatment and linkage to social services. We enhanced the original phenotype by adding additional elements (see below) to improve sensitivity and accommodate our institutional electronic health record structure, recognizing that opioid use disorder diagnostic codes are often underdocumented due to concerns about potential downstream bias in care.

Although triage-based screening for opioid use disorder is intended in our ED, it is not consistently performed, and when documented, it is typically recorded in nursing flowsheets that are not readily accessible or routinely reviewed by clinicians. Therefore, our site-adapted structured opioid use disorder phenotype triggers the clinical decision support tool if any of the following are true: one or more ICD-10 codes indicating opioid use disorder, withdrawal, or overdose in encounter (discharge and/or admission) diagnoses, problem lists, or past medical history; active prescriptions for buprenorphine or methadone preceding the index ED visit encounter; one or more positive urine toxicology for methadone, fentanyl, heroin or their metabolites; one or more addiction care team consultation notes mentioning opioid-related terms; and text recognition of specific terms (“opioid use disorder,” “opioid use disorder,” “COWS,” “heroin,” “fentanyl”) in ED clinical notes, including reason for visit free text.^8^

#### Large language model.

We used a HIPAA-compliant version of ChatGPT 4.1 (OpenAI), an out-of-the-box model not trained on UCSF data and not updated based on study inputs. All notes associated with the ED encounter (excluding student and procedure notes) were concatenated into the prompt without additional labels beyond note titles. The prompt asked the model to determine whether the patient had opioid use disorder based on the clinical documentation and was iteratively defined based on 10 representative ED encounters selected to reflect a range of clinical scenarios (details provided in Appendix E1, available at http://www.annemergmed.com). The model was instructed to return a binary classification (YES/NO) and a brief rationale in a standardized format (Figure).

#### Human reference standard.

Two board-certified emergency medicine attending physicians, one with certification in addiction medicine and toxicology (KL) and the other in clinical informatics (MM), independently and manually reviewed each patient’s entire medical record, including Care Everywhere documentation, blinded to the results of both the structured phenotype and the large language model evaluation, to determine the presence of opioid use disorder. They were deliberately given full chart access to reflect real-world clinical practice.

Because formal Diagnostic and Statistical Manual of Mental Disorders, 5^th^ Edition (DSM-5) criteria for opioid use disorder are often incompletely documented in the electronic health record, we used a simplified, clinically oriented definition intended to identify patients who would reasonably be considered for treatment in the ED. A patient was determined to have opioid use disorder if there were any of the following: 1) documented diagnosis of opioid use disorder, 2) history of opioid withdrawal occurring in the setting of nonprescribed or problematic opioid use, 3) history of opioid overdose via intentional use of an opioid, or 4) more than one episode of intentional illicit or nonprescribed opioid use. Opioid dependence was excluded. Discrepancies were adjudicated by a third attending physician (AK).

### Analysis

We used descriptive statistics to characterize ED encounters by opioid use disorder presence/absence and assessed interrater agreement using Cohen’s κ with 95% confidence intervals (CI). Stratified random sampling with the 2 strata defined by an index test is test result-based sampling.^9–12^ For the structured phenotype, the naively calculated positive predictive value and negative predictive value are unbiased, but estimating sensitivity and specificity requires weights equal to the inverse sampling fraction. For a large language model, all metrics, positive predictive value, negative predictive value, sensitivity, and specificity require inverse sampling weights. We used Stata’s survey procedures with the finite population correction to calculate the weighted estimates with 95% CI. Differences in sensitivity and specificity between tests were assessed with survey-weighted paired difference-in-proportions Wald tests.

Analyses were conducted in R version 4.4.2 and Stata version 9.5. We performed qualitative analysis of discordant cases using rapid content analysis of free-text comments documented by physician reviewers to explain their classification decisions. Two study investigators reviewed these comments and iteratively developed thematic categories to characterize sources of disagreement and misclassification. Discrepancies were resolved through discussion.

## RESULTS

From January 1, 2023 to October 17, 2024, there were 70,880 encounters by adults older than 18 years, 5,701 (8.0%) structured phenotype-positive. Of these, 201 (sampling fraction 0.0353) were randomly selected for application of the reference standard. Of the remaining 65,179 encounters that were structured phenotype-negative, 101 were randomly selected (sampling fraction 0.0015). In this sample of 302 encounters, 67 (22%) had multiple ED note types. Patient demographic characteristics are shown in Table 1.

There was strong interrater reliability (κ = 0.92; 95% CI, 0.88 to 0.97) between the 2 physicians’ classifications with 11 encounters requiring adjudication. After consensus, 117 (38.7%) encounters were classified as having opioid use disorder and 185 (61.3%) as not having opioid use disorder. The test characteristics of the structured phenotype and large language model compared with the reference standard are shown in Table 2.

Compared with the large language model, the computable structured phenotype demonstrated comparable sensitivity, 0.84 vs. 0.81 (*P* = .859), and worse specificity, 0.964 vs. 0.996 (*P*<.0001). The estimated population prevalence of opioid use disorder was 0.056 (95% CI, 0.040 to 0.07).

Qualitative analysis of discordant cases (Table 3) revealed distinct error patterns for each approach. Computable structured phenotype false positives primarily resulted from single positive fentanyl urine toxicology results reflecting unintentional exposure (eg, contamination of stimulant drugs with fentanyl) and chronic pain patients on methadone or buprenorphine, whereas false negatives occurred when relevant opioid use disorder information was documented in notes external to the health system (ie, Epic’s “Care Everywhere”; see Discussion).

## LIMITATIONS

Generalizability may be limited because this was a single-center study conducted at a tertiary care academic health system with relatively low opioid use disorder prevalence. Performance may differ across settings with different documentation practices, note availability, electronic health record structures, and ED workflows. Our reference standard was based on the determination of 2 physicians and included addiction consultation notes and external documentation via Care Everywhere—resources that may not be available across all health systems. Yet this approach reflects real-world clinical practice in the ED, where clinicians often make determinations about opioid use disorder based on available documentation rather than structured diagnostic assessments. The high interrater agreement observed in our study supports the reliability of this approach. Future research should validate these findings across different health systems and populations.

## DISCUSSION

Both the structured computable opioid use disorder phenotype and the large language model performed well for opioid use disorder identification compared with expert physician review. The structured phenotype demonstrated slightly higher sensitivity, whereas the large language model demonstrated near-perfect specificity and higher positive predictive value.

Given the low prevalence of opioid use disorder in our study population, caution is warranted when interpreting predictive values. Negative predictive value is expected to be high in low-prevalence settings, even for tests with modest performance. In this context, the high negative predictive value observed for both approaches reflects the underlying prevalence as much as test performance. However, both approaches demonstrated high specificity, supporting their ability to rule in opioid use disorder in most encounters. The large language model’s higher positive predictive value indicates fewer false positives, potentially reflecting greater ability to interpret clinical nuance within narrative documentation. This could ultimately improve targeting of resources toward patients most likely to benefit while reducing unnecessary alerts.

Prior studies have demonstrated that large language models can extract opioid use disorder-related concepts from clinical text and, in some settings, outperform earlier AI approaches for interpreting narrative documentation.^13–15^ However, emergency clinical decision support tools have traditionally relied on rule-based computable phenotypes, built from diagnosis codes, medications, laboratory results, and keywords.^6,16^ Our study directly compares an large language model with such a structured phenotype. Although the structured phenotype demonstrated slightly higher sensitivity, the large language model achieved almost perfect specificity and a higher positive predictive value. These complementary performance characteristics suggest a staged clinical decision support approach. A structured phenotype could serve as an initial step, followed by a large language model review to decrease false positives. In low-prevalence ED settings, this strategy may reduce alert fatigue while preserving case detection. Notably, this performance was achieved using documentation from a single ED visit, which is often not extensive. Thus, large language models may be particularly valuable in busy clinical environments, such as the ED, where time constraints limit comprehensive chart review and systematic human screening is difficult to sustain.

Future research should focus on refining reference standards and optimizing implementation across diverse clinical environments. Although expert agreement was high, disagreement in select cases reflects challenges in applying DSM-5 criteria in electronic health record review, where key elements may be incompletely documented, particularly when distinguishing opioid use disorder from physiologic dependence and other forms of opioid exposure. These ambiguities affect both clinicians and automated systems; rather than expecting perfection, the key question is whether large language models can achieve expert-level performance in a scalable way. In the ED, where systematic screening may not occur consistently because of time and resource constraints, a large language model that performs at or near the level of expert physician review may extend expert-level assessment to encounters where manual review is not feasible. Continued work on prompt engineering and more nuanced classification frameworks may improve model performance in ambiguous scenarios. Prospective studies incorporating standardized diagnostic criteria and multidisciplinary adjudication may further strengthen outcome definitions. Future evaluations should also incorporate longitudinal records, including outside documentation available through health information exchange (eg, Care Everywhere), and assess performance across multiple health systems and real-world workflows. Such efforts will help define how AI-assisted opioid use disorder identification can be responsibly integrated into ED-based clinical decision support.

## Supplementary Material

Appendix E1

## Figures and Tables

**Figure.**
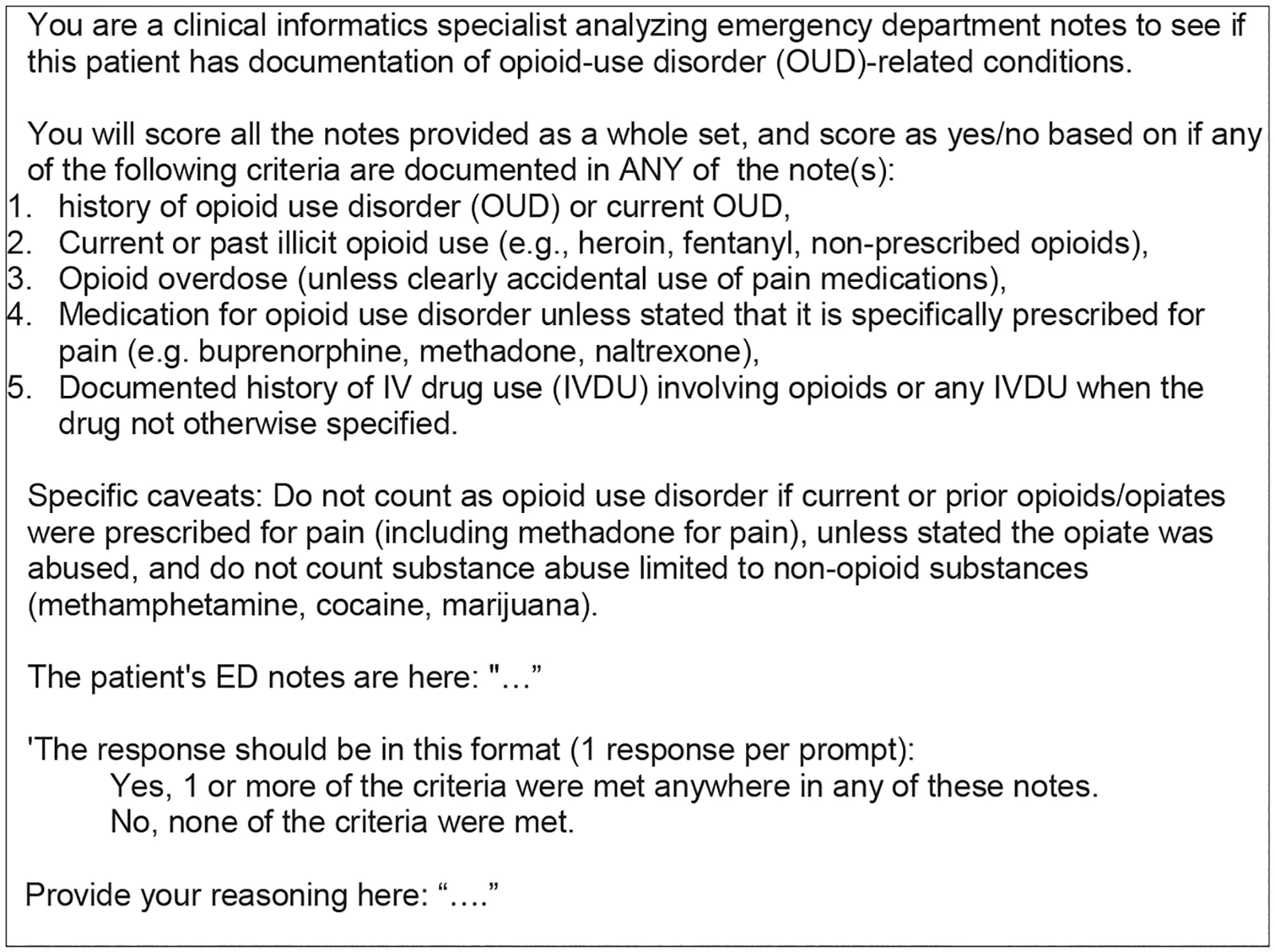
Large language model prompt to identify opioid use disorder.

**Table 1.** Patient demographic characteristics of ED encounters.

Characteristic	Total Encounters N (%)	Structured Phenotype Positive n (%)	Structured Phenotype Negative n (%)
**Overall**	302	201	101
**Age, Y, Mean (SD)**	50.4 (17.9)	49.2 (16.5)	53.0 (20.2)
**Race and Ethnicity**			
Asian	39 (12.9)	14 (7)	25 (24.8)
Black	54 (17.9)	36 (17.9)	18 (17.8)
Latinx	42 (13.9)	26 (12.9)	16 (15.8)
Multiracial	12 (4)	9 (4.5)	3 (3)
Other*	18 (6)	16 (8)	2 (2)
White	136 (45)	99 (49.3)	37 (36.6)
**Sex**			
Male	184 (60.9)	128 (63.7)	56 (55.4)
Female	114 (37.7)	69 (34.3)	45 (44.6)
Other^†^	4 (1.3)	4 (2)	0 (0%)
**Insurance**			
Medicaid	107 (35.4)	96 (47.8)	11 (10.9)
Medicare	101 (33.4)	65 (32.3)	36 (35.6)
Commercial	72 (23.8)	26 (12.9)	46 (45.5)
Other^‡^	4 (1.3)	4 (2)	0 (0%)
**Language: Interpreter Needed?**	18 (6)	7 (3.5)	11 (10.9)

* Included “Other,” “Unknown/Declined,” “Southwest Asian and North African”

† Included “Choose not to disclose,” “Declines to answer,” “Something else,” “Unknown”

‡ Included “Other” and “Worker’s Comp”

**Table 2.** Test characteristics of computable structured phenotype versus large language model

Identification Method	OUD on Manual Review	Non-OUD on Manual Review	Adjusted Sensitivity* (95% CI)	Adjusted Specificity* (95% CI)	Adjusted PPV* (95% CI)	Adjusted NPV* (95% CI)
OUD by Structured Phenotype	116	85	0.84 (0.42–0.97)	0.96 (0.96–0.97)	0.58 (0.51–0.64)^†^	0.99 (0.93–1.0)^†^
Non-OUD by Structured Phenotype	1	100				
OUD by LLM	90	9	0.81 (0.70–0.88)	0.996 (0.993–0.998)	0.92 (0.85–0.96)	0.99 (0.98–0.99)
Non-OUD by LLM	27	176				

*LLM*, large language model; *PPV*, positive predictive value; *NPV*, negative predictive value; *OUD*, opiate use disorder.

* Adjusted based on sampling weights: 1/0.035 for phenotype positive, 1/0.0016 for phenotype negative.

† Did not require adjustment because sampling was stratified by structured phenotype.

**Table 3.** Themes from discordant classifications

Classifier	False Positive	False Negative
Structured	Urine toxicology resulted positive for fentanyl once without intentional use of opioid (eg, contamination of stimulant drugs with fentanyl) Chronic pain on opioid medication (including methadone or buprenorphine)	Missed mention of overdose responsive to naloxone in Care Everywhere ED note History of “opiate withdrawal”* in Care Everywhere Cardiac arrest related to “OUD”
LLM	One suicide attempt by overdose of fentanyl without consistent use of opioids Opioid dependence in Problem List counted as OUD Counted withdrawal from kratom as opioid-like substance^†^	Missed mention of OUD on methadone as part of Care Everywhere Missed mention of overdose responsive to naloxone in Care Everywhere ED note Anesthesia Procedure Note mention of “polysubstance abuse,” including OUD OUD documented in Care Everywhere notes
Expert 1^‡^	Stimulant use mislabeled as OUD in provider notes Urine toxicology positive for fentanyl twice, but no explicit mention of intentional use of opioid	Methadone “abuse” reported by the patient’s wife misclassified as methadone maintenance Missed mention of prior heroin addiction
Expert 2^‡^	Opioid dependence falsely categorized as OUD due to explicit (incorrect) mention of “use disorder” in provider notes, despite no documented evidence of a use disorder	Missed mention in provider note of regular opioid use combined with fentanyl positive on urine drug screen Patient on buprenorphine transdermal patch for chronic pain but multiple urine toxicology results with fentanyl positivity Missed mention of prior heroin addiction and opioid withdrawal

*ED*, emergency department.

* Quotations indicate descriptions quoted from the patient’s electronic health record.

† Although kratom has opioid receptor activity, it differs pharmacologically from traditional opioids and has dose-dependent stimulant and opioid-like effects; cases with kratom-associated withdrawal were excluded to avoid misclassification.

‡ These represent discordant cases between Expert 1 and Expert 2.

## Data Availability

Deidentified data underlying this study contain sensitive protected health information and are not publicly available. Data may be made available from the corresponding author on a case-by-case basis to qualified researchers with appropriate institutional review board approval and data use agreements, in accordance with institutional and regulatory policies. All authors attest to meeting the four ICMJE.org authorship criteria: (1) Substantial contributions to the conception or design of the work; or the acquisition, analysis, or interpretation of data for the work; AND (2) Drafting the work or revising it critically for important intellectual content; AND (3) Final approval of the version to be published; AND (4) Agreement to be accountable for all aspects of the work in ensuring that questions related to the accuracy or integrity of any part of the work are appropriately investigated and resolved.

## Editor’s Capsule Summary

### What is already known on this topic

Screening for opioid use disorder is widely conducted and potentially inaccurate.

### What question this study addressed

How well did an off-the-shelf large language model (ChatGPT 4.1) identify patients with opioid use disorder compared to a more traditional structured approach?

### What this study adds to our knowledge

The large language model had similar sensitivity, slightly better specificity, and overall better positive predictive value.

### How this is relevant to clinical practice

Large language models with good accuracy might be developed for site-specific screening tasks.
