# Supplementary material for: Computable Structured Phenotype Versus Large Language Model Identification of Opioid Use Disorder Using Electronic Health Record Data
Source: Ann Emerg Med. Author manuscript; Available in PMC 2026 Jul 22. (PMC13388841; doi:10.1016/j.annemergmed.2026.05.005)
Supplement: Appendix E1 [file NIHMS2195284-supplement-Appendix_E1.docx]

**Appendix A. Large Language Model Opioid Use Disorder Identification Prompt Development**

Prompt development was conducted using a separate set of ten representative ED encounters selected to reflect a range of clinical scenarios related to opioid use and OUD. Initial prompt wording focused on identifying any mention of opioid-related substance use; early iterations resulted in false positives in cases involving prescribed opioids for acute pain or family history of OUD. The prompt was subsequently refined to clarify interpretive caveats (e.g., distinguishing prescribed opioid use for pain from misuse) and to require documentation meeting predefined clinical criteria for OUD. After refinement, the prompt was finalized and fixed prior to application to the full study cohort.

The model (OpenAI ChatGPT-4.1) was accessed via R (v4.4.0) using the OpenAI API. Each ED encounter was processed independently in a single request using deterministic inference (temperature = 0). No conversational memory was retained between encounters. The model was instructed to return a binary classification (YES/NO) and a brief rationale in a standardized format. Model outputs were stored verbatim and were not manually edited prior to analysis.
